# Improvements in the preparation of phosphate for oxygen isotope analysis from soils and sediments

**DOI:** 10.1371/journal.pone.0204203

**Published:** 2018-09-20

**Authors:** Zifu Xu, Tao Huang, Xijie Yin

**Affiliations:** 1 School of Resources and Environmental Engineering, Anhui University, Hefei, China; 2 Third Institute of Oceanography, State Oceanic Administration, Xiamen, China; Rothamsted Research, UNITED KINGDOM

## Abstract

In contrast to the successful preparation of phosphate for oxygen isotope analysis from water samples, there are still a series of problems for similar analyses from soils and sediments. Here, we improved and optimized the methods of silver phosphate preparation for oxygen isotope analysis from soils and sediments. During our preparations, organic matter was removed by sodium hypochlorite and XAD-2 resin, while the impurities of elemental silver and its oxide were removed by rapid microprecipitation and ammonium phospho-molybdate and magnesium ammonium phosphate. The total organic carbon and total nitrogen in the prepared silver phosphates from soils and sediments were 0.226±0.033% and 0.030±0.0059% (n = 7), 0.217±0.053% and 0.034±0.0120% (n = 9), respectively, indicating a high removal efficiency of organic matter. We confirmed that adding citric acid during rapid microprecipitation would introduce the impurity of elemental silver, which could be removed by ammonia recrystallization. The pH range of solutions for rapid microprecipitation was optimized at 7.0‒7.5. Results of X-ray Diffraction and stable oxygen isotope analyses showed that the improved method could obtain high pure silver phosphate from soil and sediment samples without oxygen isotope fractionation. This improved procedure provides a foundation for biogeochemical studies on phosphorus in soil and lacustrine environments by using phosphate oxygen isotopes.

## Introduction

Phosphorus is present in the form of phosphate in natural environments. Phosphorus has only one stable isotope ^31^P, therefore, phosphate oxygen isotopes can be used to study the geochemical cycle of phosphorus [1]. In addition, phosphate maintains its oxygen isotope signal under earth surface temperatures and pH conditions without biochemical interference [2–4], so it is increasingly recognized as a powerful proxy for tracing the geochemical cycle of phosphorus [5–8].

Phosphate oxygen isotope analysis is performed primarily on silver phosphate which is prepared from natural samples [9]. Thus, the purity of the prepared silver phosphate determines its oxygen isotope ratios. Importantly, it is necessary to remove impurities such as organic matter, elemental silver and silver oxide through a series of purification steps during sample preparation [3]. For example, the oxygenated impurities could change the phosphate oxygen isotope signal directly [10]. Impurities of elemental silver, excessive chlorides and metal cations could also affect the final phosphate oxygen isotope signal indirectly [10]. To remove these impurities, some methods include CePO_4_ precipitation, XAD-2 resin separation and cation exchange resin separation during sample preparation [2, 10–14]. For example, McLaughlin et al. prepared high purity silver phosphate from water samples by using these methods [13].

Recently, the phosphate oxygen isotope methodology has been applied to determine the sources, migrations and transformations of phosphorus in soil and lacustrine environments [15–20]. However, in soils and sediments the complex matrix and high levels of organic matter and metal cations, the methods used for water samples may not always be suitable [21, 22], such as the method of cerium phosphate. For example high metal cations in soils and sediments would hinder formation of CePO_4_ and block the process of purification. In addition, Tamburini et al. [11] have pointed out that the organophosphates or pyrophosphates in soils and sediments would be hydrolyzed during the preparation methods of McLaughlin et al. [13]. Tamburni et al. [11] prepared silver phosphate successfully from soils by using a classical phosphate purification method: ammonium phospho-molybdate and magnesium ammonium phosphate (APM-MAP). However, a recent study [23] indicated that a large amount of residual organic matter was found in the prepared phosphates using these methods. In addition, the use of hydrogen peroxide could affect the oxygen isotope ratio of the prepared phosphate [24, 25]. In fact, many studies have problems with the removal of organic matter during sample preparations [18, 21, 24, 25], though numerous different methods have been applied to try and resolve this issue.

In addition to the issue of organic matter, silver oxide was found in the silver phosphate when using the method of microprecipitation to prepare phosphate [23]. Rapid microprecipitation has been widely used in silver phosphate precipitation [26], though the pH range of its solution system is controversial [27]. An improved rapid microprecipitation has been proposed by Mine et al. [27] to resolve the problems of incomplete precipitation of phosphate and the production of silver oxide contaminants during the preparation of silver phosphate from bioapatites, though it is not suitable for samples of soils and sediments. In addition, we found elemental silver would be produced during preparation by rapid microprecipitation and APM-MAP in our study. Since elemental silver can be oxidized easily to silver oxide, it affects the final oxygen isotope values.

To resolve the above problems, we propose here an improved technological procedure and method for the preparation of acid-soluble phosphate for oxygen isotope analysis from soils and sediments, based on the methods of Tamburni et al. [11] and Zhang et al. [23]. We removed the organic matter by using sodium hypochlorite and XAD-2 resin, and the inorganic impurities (elemental silver) by rapid microprecipitation and ammonia recrystallization. We optimized the pH range of the silver phosphate precipitation system during rapid microprecipitation and examined the fractionation of the oxygen isotope during the steps of our extraction and purification. This study improves the methodology or geochemical studies on phosphorus in soils and sediments by using phosphate oxygen isotopes.

## Materials and methods

### Ethics statement

- **We state clearly that no specific permissions were required for these locations/activities, and provide details on why this is the case**.
- **We confirm that the field studies did not involve endangered or protected species.**

We stated the geographical coordinates of the soil samples in Table 1.

**Table 1:** TOC, TN and IP in the samples of this study.

Sample	Sample type	TOC %	TN %	IP mg/kg	Latitude	Longitude
1	Farmland soils	3.598	0.397	3395.7	31°31'40.7"N	117°14'03.5"E
2	Farmland soils	2.671	0.174	902.2	31°33'09.8"N	117°10'36.5"E
3	Farmland soils	1.814	0.241	462.7	31°32'53.3"N	117°05'17.4"E
4	River sediments	2.918	0.304	1507.6	31°42'31.1"N	117°24'18.5"E
5	River sediments	1.377	0.155	267.9	31°53'00.5"N	117°12'50.0"E
6	River sediments	2.647	0.164	574.1	31°52'34.2"N	117°17'00.4"E

We stated the source for all materials and reagents used in this study in reagents and samples section.

### Reagents and samples

The reagents used in this study include XAD-2 Resin (Amberlite, 40–60 mesh), AG 50W-X8 Resin (BioRad, H^+^ type, 100‒200 mesh), Millipore polycarbonate membrane (0.2μm), sodium hypochlorite (GR, Sinopharm), silver nitrate (GR, Sinopharm), ammonia (GR, Sinopharm), nitric acid (GR, Sinopharm), ammonium molybdate (AR, Sinopharm), citric acid (AR, Sinopharm), magnesium chloride (AR, Sinopharm) and silver phosphate (99%, Alfa Aesar).

The soil and sediment samples used here were collected from farmlands and rivers along the Chaohu river basin, China. Samples were freeze-dried and sieved at < 2 mm before chemical extraction. Table 1 provides the coordinates of the samples and the contents of Total Organic Carbon (TOC), Total Nitrogen (TN) and acid soluble inorganic phosphorus (IP).

### Methods

#### Preparation of silver phosphate

Step 1: Removal of organic matter by sodium hypochlorite

We weighed 5.0 g (for the samples with low phosphorus content, the sample sizes should be increased appropriately) of soil or sediment per sample and placed them into a centrifuge tubes with 50 ml sodium hypochlorite (acidified to pH = 8 with 0.5 mol/L HCl). The mixture was agitated for 24 hours at 180 rpm at room temperature, centrifuged at 8000 rpm for 10 min, and washed with Milli-Q water 4‒6 times to neutral pH. The supernatant was discarded and the residue (referred to henceforth as Residue 1) was dried at 40°C.

Step 2: Extraction of phosphate

Residue 1 was placed in a centrifuge tubes with 120 ml HCl (1 mol/L), agitated for 24 h at 180 rpm at room temperature, and centrifuged for 10 min at 8000 rpm. The supernatant is now referred to as Solution 1.

Step 3: Collection of phosphate

Phosphate was collected by MAGIC co-precipitation: the pH of Solution 1 was adjusted to pH = 10 with 5 mol/L NaOH to form Mg(OH)_2_ precipitate (due to the high concentrations of heavy metals in some soil and sediment samples, red metal hydroxides associated with Mg(OH)_2_ can be produced). The precipitate was allowed to settle for 6 h and was then centrifuged for 10 min at 8000 rpm to remove the supernatant. The residue is now referred to as Residue 2.

Step 4: Removal of organic matter by XAD-2 resin

Residue 2 was dissolved in 15 ml nitric acid (5 mol/L) and passed through a column packed with XAD-2 resin at a flow rate of 1 ml/min. A large amount of organic matter was adsorbed to the resin and the residual solution collected referred to as Solution 2.

Step 5: Formation and dissolution of APM-MAP [11]

Twenty-five ml NH_4_NO_3_ solution (4.2 mol/L) and 40 ml ammonium molybdate solution (10 g of (NH_4_)_6_Mo_7_O_24_)·4H_2_O and 90 ml of Milli-Q water) were added to Solution 2 and the combined solution was placed in a flask and agitated in a water bath at 50°C for 18h. Yellow ammonium phosphomolybdate were precipitated during the mixing process. Filtered the residue onto a 0.2 micron polycarbonate membrane, washed with NH_4_NO_3_ (0.6 mol/L) and transferred to a 50 ml conical flask. Fifty ml of citric acid-aqueous buffer solution (10 g citric acid, 140 ml concentrated NH_4_OH, 300 ml Milli-Q water) was added to the conical flask to dissolve the precipitate and it is referred to as Solution 3.

The pH of Solution 3 was adjusted to 8‒9 with concentrated ammonia and 25 ml of magnesia solution was added (50 g MgCl_2_·6H_2_O and 100 g NH_4_Cl dissolved in 500 ml H_2_O, acidified to pH = 1 with 12 mol/L HCl and the volume adjusted to 1:1 with Milli-Q water). After a white magnesium ammonium phosphate precipitate was formed and filtered onto a 0.2 micron polycarbonate membrane and rinsed 2‒3 times with 1:20 ammonia. The precipitate was transferred to a 50 ml conical flask dissolved by HNO_3_ and referred to Solution 4 (the precipitates were dissolved by 3–4 ml 0.5 mol/L HNO_3_, then 10–20 mL Milli-Q water was added to ensure that [H^+^] in the solution was less than 0.2 mol/L).

Step 6: Removal of cations

Solution 4 was mixed with cation exchange resin and agitated at room temperature for 24 h to remove magnesium ions. The sample was separated from the resin by a chromatography column with a sand core. The chromatography column was washed with Milli-Q water 2‒3 times, and the filtrate collected as Solution 5.

Step 7: Formation of silver phosphate

The pH of Solution 5 was adjusted to 7.0‒7.5 by concentrated ammonia or nitric acid. The yellow silver phosphate was precipitated by adding silver nitrate (8 g AgNO_3_, 16 ml Milli-Q water); yellow precipitates were covered by gray-black impurity (elemental silver) gradually with time. The formation of silver phosphate decreased the pH of the solution over time; therefore it was necessary to adjust the pH continuously using concentrated ammonia maintain a pH at 7.0‒7.5.

Step 8: Purification of silver phosphate [23]

The silver phosphate was collected from the solution by filtration through a 0.2 micron filter, washed several times to remove silver ions completely by using Milli-Q water, placed in a 50 ml polypropylene tube and dried at 50°C. The silver phosphate was then dissolved by ten ml concentrated aqueous ammonia and collected through a 0.2 micron polycarbonate membrane. During this filtration, gray-black impurities (elemental silver) were removed as they would not dissolve in aqueous ammonia. The filterable solution was collected in a 50 ml polypropylene tube and placed in an oven at 40°C and the bright yellow silver phosphate was precipitated after the ammonia evaporated within half an hour. The silver phosphate was then filtered with a 0.2 micron polycarbonate membrane, washed using Milli-Q water 2 or 3 times and dried at 40°C.

#### Analysis of TOC, TN and IP

The TOC and TN in the prepared silver phosphates were measured by vario EL III (Elementar, Germany) with a relative error of 0.1%. The acid soluble IP in sediments and soils were measured by the ammonium molybdate spectrophotometric method [28].

#### Analysis of X-ray Diffraction (XRD)

The dried prepared silver phosphate samples were passed through a 20 mesh sieve prior to mineral analyses by X-ray diffractometer (X’Pert PRO, PANalytical).

#### Analysis of stable oxygen isotope

The stable oxygen isotopes of silver phosphate were analyzed by an elemental analyzer-stable isotope ratio mass spectrometer (Flash EA 1112 HT-Mat 253, Thermo). After closing the oxygen injection, silver phosphate was compacted into a silver boat and reacted with graphite to form CO at 1400°C. The generated CO was transferred into a chromatographic column with He gas (90 ml/min) for the separation from trace N_2_ in the carrier gas under 85°C and finally detected by stable isotope mass spectrometer. In addition, pure silver phosphate (USGS 1, *δ*^18^O = 9.8‰) was used as the working standard. The instrument precision was ±0.30‰ for *δ*^18^O. Stable isotope results are presented in *δ* (‰) and expressed relative to the standard mean ocean water (SMOW) for *δ*^18^O according to the equation: *δ* (‰) = [(R_sample_ − R_standard_)/R_standard_] * 10^3^, where *δ* (‰) represents the *δ*^18^O value, R_sample_ is the isotopic ratio of the sample, and R_standard_ the isotopic ratio of SMOW.

#### Statistical test

To assess the significance of the difference in elemental and stable isotope data between different treatments or methods, we performed two-step statistical tests in SPSS 24.0. We use the nonparametric one-sample K-S test to verify if the data meet a normal distribution. If so, we performed parametric independent samples t-tests at a significance level of p < 0.05.

## Results and discussion

### Improvements for the removal of impurities

#### Organic matter

**Fig 1:**
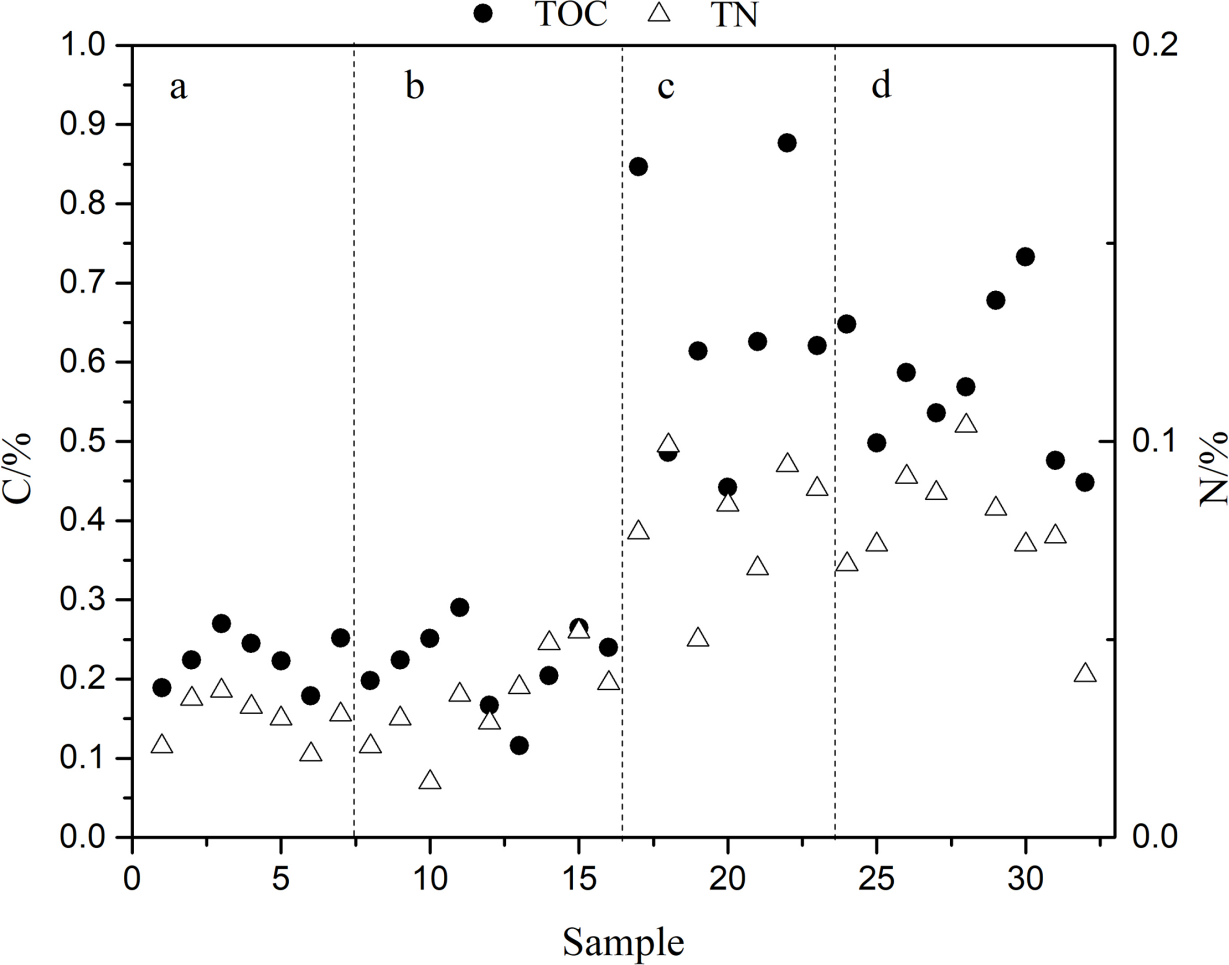
TOC and TN in the prepared silver phosphates which were purified by different methods in this study. (a) Soil samples treated by sodium hypochlorite. (b) Sediment samples treated by sodium hypochlorite. (c) Soil samples without treatment of sodium hypochlorite. (d) Sediment samples without treatment of sodium hypochlorite.

**Fig 2:**
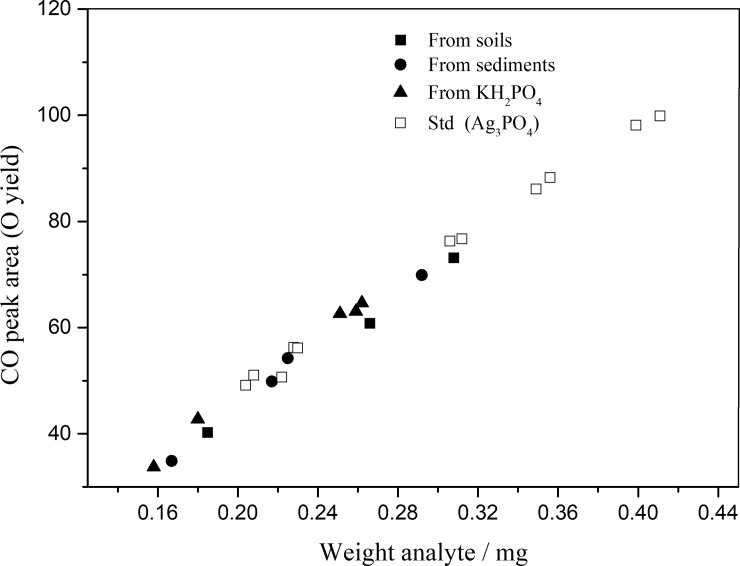
Weight of analyte plotted against the area of the CO peak for the prepared silver phosphates treated by sodium hypochlorite and the standard silver phosphates.

Usually, it is difficult to remove the organic matter efficiently from soils and sediments during sample preparation by any single method [18, 21, 24, 25]. In our approach we removed organic matter by a multi-step procedure. Firstly, prior to acid extraction, organic matter was removed by sodium hypochlorite (2.5%), which by oxidation can reduce organics without affecting inorganic constituents in soils and sediments [29, 30]. Secondly, during the purification of extracted phosphate, residual organic matter was removed by XAD-2 resin and ammonia recrystallization. To determine the removal efficiency of organic matter, the TOC and TN of the prepared silver phosphates were measured at 0.226±0.033% and 0.030±0.0059% (n = 7), 0.217±0.053% and 0.034±0.0120% (n = 9), respectively (Fig 1 and S1 Table); samples without this treatment of sodium hypochlorite measured at 0.645±0.164% and 0.081±0.0167% (n = 7), 0.574±0.096% and 0.078±0.0174% (n = 9). These results indicate a high removal efficiency of organic matter by treatment with sodium hypochlorite and XAD-2 resin. To further investigate the removal of organic matter, the CO peak yield of the silver phosphate prepared from soils, sediments, pure KH_2_PO_4_ and the Ag_3_PO_4_ standards was also measured by an element analyzer. All samples and the Ag_3_PO_4_ standards lie along a regression line (Fig 2 and S2 Table), indicating pure Ag_3_PO_4_.

#### Elemental silver

**Fig 3:**
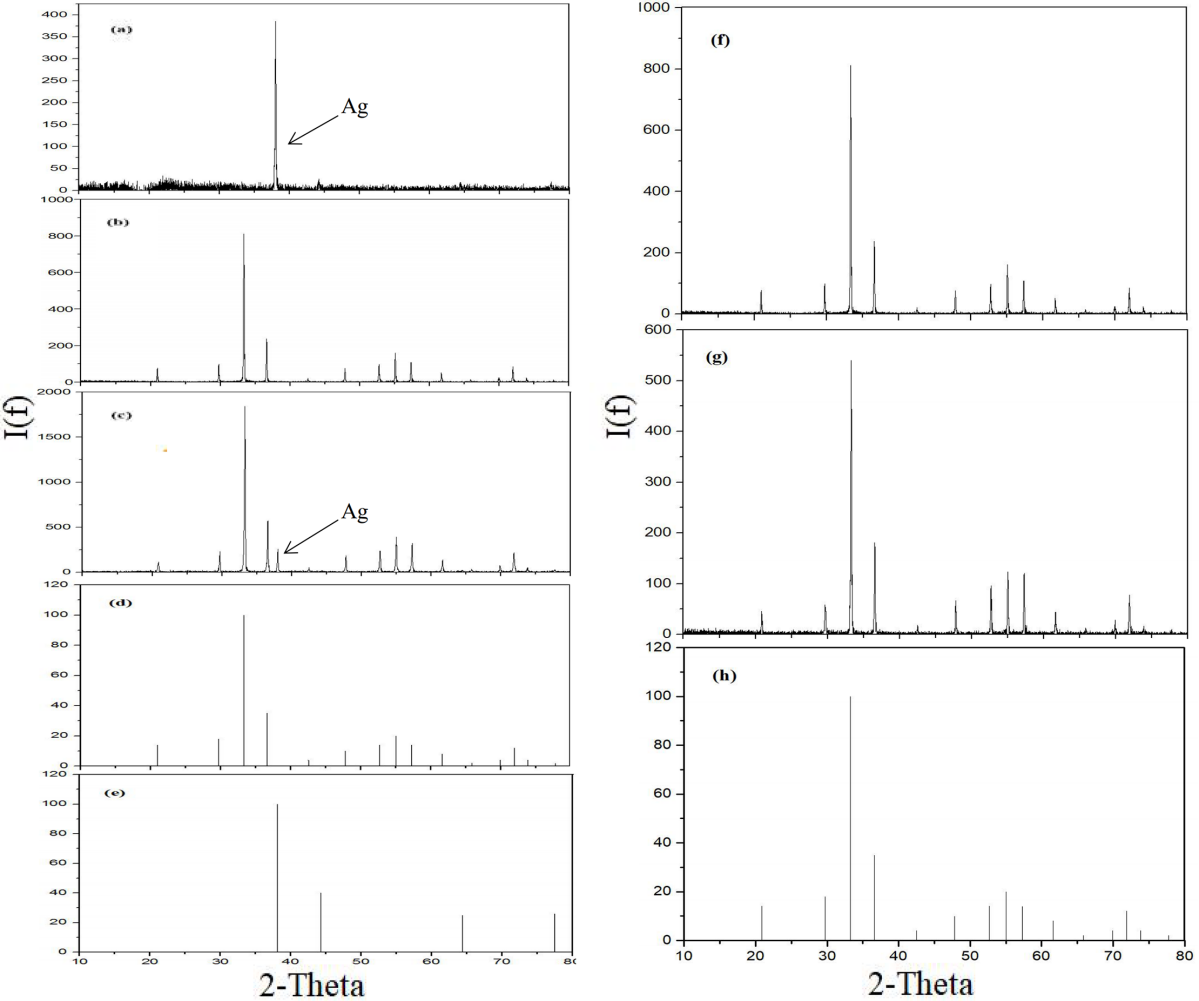
XRD spectrums of the silver phosphates by different treatments. The arrows in the graphs indicate the peak of the elemental silver. (a) graph showing the characteristic peak of elemental silver, which indicates that the gray and black impurity obtained by filtration is elemental silver. However, in the graph (b), the peak of the elemental silver obviously disappears; (c) graph indicating that there are obvious peaks of elemental silver in silver phosphate precipitation, which demonstrates that silver elements are mixed in silver phosphate. (a): (filtered) the gray-black impurity, (b): prepared from KH_2_PO_4_ with dissolving APM by citric acid-ammonia, (c): prepared from KH_2_PO_4_ with dissolving APM by aqueous ammonia, (f): prepared from soil after aqueous ammonia recrystallization, (g): prepared from sediment after aqueous ammonia recrystallization), the standard of silver phosphate ((d) and (h), numbered 06–0505) and the standard of elemental silver ((e): numbered 64–2871).

**Fig 4:**
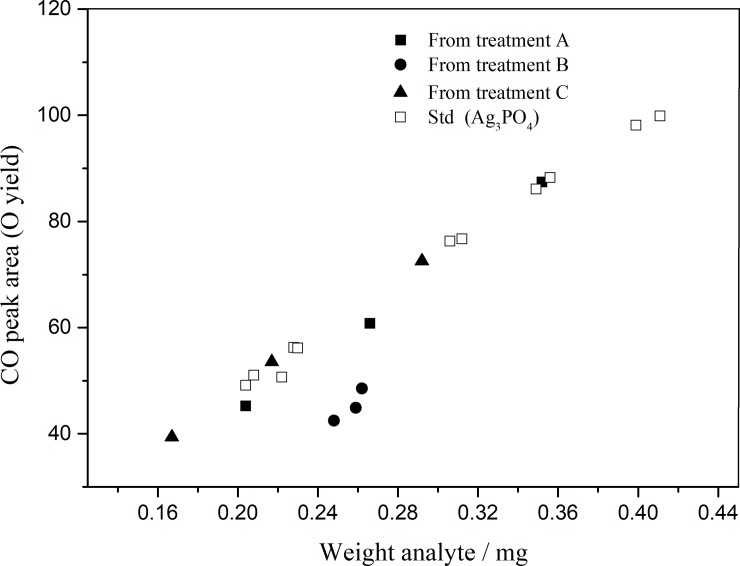
Weight of analyte plotted against the area of the CO peak for the prepared silver phosphate treated by each treatment.

The method of APM-MAP combined with rapid microprecipitation is applied primarily in the purification and precipitation of phosphates from soils and sediments [11, 13, 23]. However, it was found that the silver phosphate was covered with a gray-black impurity of elemental silver (determined by XRD, Fig 3A) during its precipitation by rapid microprecipitation using APM-MAP to purify phosphate, whether it was prepared from soils, sediments, or pure KH_2_PO_4_. The impurity of elemental silver can be separated from silver phosphate by aqueous ammonia. To explore the production mechanism of elemental silver, we repeated the preparation of silver phosphate from pure KH_2_PO_4_ with different treatments (Table 2). For treatment A, KH_2_PO_4_ and silver ammonia solution were mixed directly, and silver phosphate was obtained without gray-black impurity. For treatment B, a parallel KH_2_PO_4_ was purified through the APM-MAP steps and a gray-black impurity was still observed during the precipitation of silver phosphate. For treatment C, in the same conditions, a gray-black impurity was not observed when the citric acid-ammonia was replaced by 1:1 aqueous ammonia. These observed phenomena were supported by the results of an XRD analysis (Fig 3B and 3C), and suggest that the impurity of elemental silver was introduced by the citric acid. The oxygen isotope ratios of the prepared silver phosphates from three different treatments (Table 2) indicated a significant contamination from the formation of elemental silver for the final isotope values. To further determine the purity of prepared phosphate, the CO peak yield of silver phosphates prepared by different treatments and the Ag_3_PO_4_ standards were measured by an element analyzer. The CO peak yield of silver phosphates from treatment A, C and the Ag_3_PO_4_ standards lie along a regression line (Fig 4 and S3 Table), while those from treatment B deviate from the regression line; indicating contaminated silver phosphates from treatment B.

**Table 2:** Study on mechanism of gray black precipitation (n = 3).

Treatment	KH_2_PO_4_	APM^a^-MAP	NaOH^b^	AgNO_3_	Impurity	*δ*^18^O_p_	σ
A	1 ml	-	-	0.6 ml	-	5.87	0.17
B	1 ml	√^c^	√	0.6 ml	√	12.41	0.18
C	1 ml	√^d^	√	0.6 ml	-	5.82	0.20
	PO_4_^3-^ source	Purification step	pH adjustment	Ag source	Phenomenon		

Note: [KH2PO4] = 50 mM, [HNO3] = 5 M, [NaOH] = 2 M, [AgNO3] = 1.1 M.

a Before the generation of APM, HNO3 was used to adjust the pH of solution to 1.0.

b NaOH was used to adjust the pH of solution from 5.0 to 7.0.

c MAP was dissolved by citric acid-ammonia.

d MAP was dissolved by 1:1 aqueous ammonia.

According to our experiment, for soil and sediment samples with high levels of iron, it was found that reddish-brown precipitates were formed when using aqueous ammonia to dissolve the APM, and therefore the purification was blocked. This results because APM adsorbs iron which could form ferric hydroxide at solution when the lattice structure of APM is broken by ammonia water [31]. There is no ferric hydroxide precipitate as we used citric acid-ammonia to dissolve the APM, since the acidic and reductive conditions from citric acid prevent this. Thus, it is necessary to use citric acid to dissolve the APM [11].

We found that the impurity of elemental silver introduced by citric acid during APM-MAP and rapid microprecipitation can be removed by aqueous ammonia recrystallization because the silver phosphate can be dissolved in aqueous ammonia while the impurity of elemental silver cannot. By using this technological procedure (aqueous ammonia recrystallization [23]), the impurity of elemental silver introduced during the APM-MAP was removed in our preparations from soils and sediments (Fig 3F and 3G).

#### Silver oxide

The rapid microprecipitation method proposed by Dettman et al. [26] is used primarily to form silver phosphate during sample preparation. This method includes adding silver nitrate and adjusting the pH to 8.0 to complete the precipitation of silver phosphate [26]. In our rapid microprecipitation, however, a gray impurity of silver oxide (determined by XRD) was found on the formed silver phosphate when using the procedure of Dettman et al. [26], no matter the phosphate was prepared from soils, sediments, or pure KH_2_PO_4_. As the mechanism for the formation of silver oxide, we speculate that it is introduced by the high pH of the solutions and designed an experiment to test this. The results indicate that, since the pH of the system will decrease during the process of silver phosphate production (a decrease in pH is related to the concentration of phosphate), it is necessary to adjust the pH by adding nitric acid and ammonia water during the production of silver phosphate. It was found that the formed silver phosphate was covered by gray impurity in the solutions of pH>7.5 during the rapid microprecipitation (Table 3). This result is likely due to silver ion and hydroxide ion forming silver hydroxide, which is then easily transformed to silver oxide, while it is difficult to precipitate silver phosphate when the pH is below 6.5 (Table 3). In a recent study, incomplete precipitation of phosphate with associated isotopic fractionation was observed in solutions at pH≤7.0 during rapid microprecipitation, while the production of silver oxide was observed if the pH was elevated to above neutral [27]. Therefore, we optimized the pH range at 7.0‒7.5 for the solution for rapid microprecipitation.

**Table 3:** Reaction phenomena in forming silver phosphate at different pH levels by rapid microprecipitation.

pH	Reaction phenomenon
5.5	No precipitate formed
6.0	A little of yellow solids suspended and precipitated gradually after a long time
6.5	A large amount of yellow precipitates formed
7.0	A large amount of yellow precipitates formed
7.5	A large amount of yellow precipitates formed
8.0	Yellow precipitates formed and covered by gray impurity (silver oxide) gradually
8.5	Yellow precipitates formed and covered by gray impurity (silver oxide) gradually

Zhang et al. [23] proposed using ammonia recrystallization to purify the prepared silver phosphate from soils. We found that it is necessary to restrict the time of ammonia recrystallization as a gray impurity of silver oxide (determined by XRD) would be introduced in this recrystallization over time. This recrystallization occurs because the silver ions are excessive during the ammonia recrystallization, and they will produce silver hydroxide and thus silver oxide with hydroxide ions after all of the phosphates are precipitated to Ag_3_PO_4_. After numerous experimental tests with our samples, it was found that the silver oxide was formed regularly after 30 minutes as the silver phosphate precipitated. Therefore, it was necessary to completely filter out the silver phosphate as they precipitate and before they form into silver oxide during the purification by ammonia recrystallization.

### Oxygen isotope fractionation

**Fig 5:**
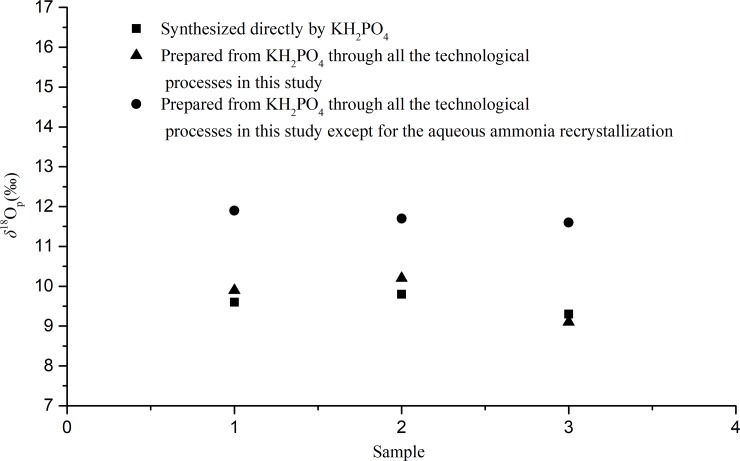
Stable oxygen isotope values of the prepared silver phosphate by different methods.

Different pretreatments of soil samples include storage, preparation and extraction that can lead to different levels of phosphate oxygen isotopic fractionation [32]. To detect if there is any fractionation of oxygen isotopes during our preparations, we measured the oxygen isotope ratios of the prepared silver phosphate from two different treatments. The first treatment was synthesized directly by KH_2_PO_4_ and silver nitrate (*δ*^18^O_p-a_); the other was prepared from the same KH_2_PO_4_ through by applying all the technological processes as proposed in this study (*δ*^18^O_p-b_). The two treatment results of *δ*^18^O_p-a_ and *δ*^18^O_p-b_ are 9.70‰ (n = 3, σ = 0.31‰) and 9.67‰ (n = 3, σ = 0.47‰), respectively (Fig 5 and S4 Table). To assess the significance of the difference in oxygen stable isotope ratios between these two treatments, we performed a homogeneity test and mean comparison test with a significant level α = 0.05, F_0.975_ (3, 5) < F = 4.813 < F_0.025_ (3, 5), t = 0.1004 < t_0.025_ (8). The results indicate that there was no significant difference between these two variances and the mean values, demonstrating that there is no oxygen isotope fractionation during our technological process.

In addition, we also measured the stable oxygen isotope ratio of the synthetic silver phosphate from KH_2_PO_4_ which was purified through the processes of APM-MAP and rapid microprecipitation, but without the aqueous ammonia recrystallization (*δ*^18^O_p_). There was a significant difference in the means and insignificant difference in the variances between *δ*^18^O_p_ and *δ*^18^O_p-a_ (*δ*^18^O_p_ = 11.84 ‰ (n = 3, σ = 0.31 ‰); *δ*^18^O_p-b_ = 9.67 ‰ (n = 3, σ = 0.47 ‰)) (α = 0.05, F_0.975_(3, 5) < F = 2.594 < F_0.025_(3, 5), t = 16.250 > t_0.025_(8)). These results further confirm that the impurity of elemental silver introduced during the process of APM-MAP and rapid microprecipitation have significantly altered the final phosphate oxygen isotope signal. Again, it is necessary to remove the impurity of elemental silver by the method of aqueous ammonia recrystallization for sample preparation.

### Recovery of phosphate

The recovery of phosphate is one of the important parameters for the process of its extraction, preparation and purification because high recovery rate could decrease the requirements of sample size, especially for those with low levels of phosphate. To determine the recovery rate of the entire preparation process, we prepared an accurate amount of silver phosphate from the soil and sediment samples, measuring the content of phosphate in every major step. The calculated results show an acceptable total recovery of phosphorus at 61.03% (Table 4).

**Table 4:** Recovery of phosphate (sample size: n = 9).

Step	Recovery	Average	Standard variance
Step 1: Sodium hypochlorite	88.4%‒93.2%	89.89%	1.23%
Step 3: Collection	91.6%‒95.7%	93.94%	1.94%
Step 4: XRD-2	98.1%‒98.9%	98.42%	0.05%
Step 5: APM-MAP	82.5%‒85.9%	84.21%	1.47%
Step 6: Removal of cations	92.4%‒95.5%	93.01%	1.26%
Step 7–8: Purification and precipitation	93.4%‒95.4%	94.21%	0.84%
Total recovery	56.8%‒68.6%	61.03%	4.80%

## Conclusions

1. High removal of organic matter and acceptable recovery of silver phosphate from soils and lacustrine sediment samples was obtained using our improved technological processes.
2. The gray-black impurity of elemental silver during the APM-MAP and rapid microprecipitation process was introduced by citric acid, which can be removed by aqueous ammonia recrystallization.
3. To avoid the impurity of silver oxide, the pH of solutions for rapid microprecipitation was optimized at 7.0‒7.5.
4. There is no oxygen isotope fractionation of the phosphate during the entire process of sample preparation in our study.
5. The improved procedure and methods proposed in this study provide a foundation for the geochemical study of phosphorus in soils and lacustrine environments by using acid-soluble phosphate oxygen isotope analysis. However, more analysis should be performed in future to study that if this procedure would work with a sequential extraction of different phosphate pools.

## Supporting information

S1 Table TOC and TN in the prepared silver phosphates which were purified by different methods in this study. (a): Soil samples treated by sodium hypochlorite. (b): Sediment samples treated by sodium hypochlorite. (c): Soil samples without treatment of sodium hypochlorite. (d): Sediment samples without treatment of sodium hypochlorite.
(XLS)

S2 Table Weight of analyte plotted against the area of the CO peak for the prepared silver phosphates treated by sodium hypochlorite and the standard silver phosphates. (XLS)

S3 Table Weight of analyte plotted against the area of the CO peak for the prepared silver phosphate treated by each treatment. (XLS)

S4 Table Stable oxygen isotope values of the prepared silver phosphate by different methods. (a): Prepared from KH_2_PO_4_ through all the technological processes in this study except for the aqueous ammonia recrystallizatione. (b): Prepared from KH_2_PO_4_ through all the technological processes in this study. (c): Synthesized directly by KH_2_PO_4_. (d): Std (Ag_3_PO_4_).
(XLS)
